# In ovo serial skeletal muscle diffusion tractography of the developing chick embryo using DTI: feasibility and correlation with histology

**DOI:** 10.1186/1471-213X-12-38

**Published:** 2012-12-26

**Authors:** Zien Zhou, Zachary DelProposto, Lianming Wu, Jianrong Xu, Jia Hua, Yan Zhou, Yongquan Ye, Zishu Zhang, Jiani Hu, E Mark Haacke

**Affiliations:** 1 Department of Radiology, Renji Hospital, Shanghai Jiaotong University School of Medicine, Shanghai, China; 2 Department of Radiology, Henry Ford Hospital, Detroit, MI, USA; 3 Department of Radiology, Wayne State University, Detroit, MI, USA

**Keywords:** Magnetic resonance imaging, Diffusion tensor imaging, Skeletal muscle, Chick embryo

## Abstract

**Background:**

Magnetic resonance imaging is a noninvasive method of evaluating embryonic development. Diffusion tensor imaging (DTI), based on the directional diffusivity of water molecules, is an established method of evaluating tissue structure. Yet embryonic motion degrades the in vivo acquisition of long-duration DTI. We used a dual-cooling technique to avoid motion artifact and aimed to investigate whether DTI can be used to monitor chick embryonic skeletal muscle development in ovo, and to investigate the correlation between quantitative DTI parameters fractional anisotropy (FA) and fiber length and quantitative histologic parameters fiber area percentage (FiberArea%) and limb length.

**Results:**

From 84 normally developing chick embryos, 5 were randomly chosen each day from incubation days 5 to 18 and scanned using 3.0 Tesla magnetic resonance imaging. A dual-cooling technique is used before and during imaging. Eggs were cracked for making histological specimen after imaging. 3 eggs were serially imaged from days 5 to 18. We show that skeletal muscle fibers can be tracked in hind limb in DTI beginning with incubation day 8. Our data shows a good positive correlation between quantitative DTI and histologic parameters (FA vs FiberArea%: r= 0.943, p<0.0001; Fiber_length vs Limb_length: r=0.974, p<0.0001). The result of tracked fibers in DTI during incubation corresponds to the development of chick embryonic skeletal muscle as reported in the literature.

**Conclusion:**

Diffusion tensor imaging can provide a noninvasive means of evaluating skeletal muscle development in ovo.

## Background

Animal models play a central role and are essential to basic medical research. Having an established pedigree of use in developmental biology, transplantation research, and cancer research, the chick embryo provides an in vivo model which is both accessible and economical, as it contains all components necessary for development, excepting heat and oxygen [1]. Pluripotent stem cells lines have been derived from various sources, including chicken [2]. Using molecular methods to identify and characterize cell surface markers and enzymatic activity, evaluation of the stem cell differentiation process is an essential step towards successful therapeutic use [3]. Most molecular imaging methods, and traditional imaging methods such as microscopy, require embryonic sacrifice for ex vivo analysis. Magnetic resonance imaging (MRI) allows embryonic imaging without sacrifice, and has been used in avian embryo analysis, primarily for evaluating anatomy [4-8]. Advanced MRI techniques, for example, diffusion-weighted imaging (DWI), diffusion tensor imaging (DTI), magnetic resonance spectroscopy, and blood-oxygen level dependent imaging (BOLD) can be used to obtain structural, metabolic, and functional information in addition to anatomic information [9,10]. However, anatomic imaging techniques and, in particular, advanced imaging techniques are challenging to implement in embryonic models due to embryonic motion [5,6,10].

Diffusion tensor imaging (DTI) is a method of observing the displacement distribution of water molecules present within an imaging voxel, allowing evaluation of tissue structure at a scale below the spatial resolution of MRI [11]. Fractional anisotropy (FA) is a DTI-derived measurement of tissue structural anisotropy ranging from 0 to 1. If the diffusion is isotropic (e.g., unrestricted, like a drop of ink diffusing in the water), the FA value is 0. If the diffusion is along a single axis (like a drop of water diffusing along a line), the FA value is 1. Tracked fiber length is another DTI-derived measurement which represents the average length of a tracked fiber-like tissue structure. Evaluation of the displacement distribution provides insight into the structure and organization of tissues, and DTI is an established method of studying the microstructure of neural tissue and skeletal muscle [12]. A disadvantage of DTI for in ovo imaging use is the long imaging duration and consequent data degradation due to subject motion. Previous work has shown that MR anatomic imaging, including real-time cardiac imaging using rapid sequence imaging, is possible with embryonic anesthesia [6-8,10]. More recently, it has been shown that cooling chick embryo from incubation day 12 prior to MR imaging allows noninvasive assessment by reducing motion artifact, without significantly affecting normal development [5]. The purpose of this study is to show that DTI can be used to monitor the development of skeletal muscle in chick embryonic hind limb using a dual-cooling technique, and investigate whether serial skeletal muscle tractography in DTI correlates with ex vivo histology from day 8 of incubation through hatching.

## Results

**Figure 1:**
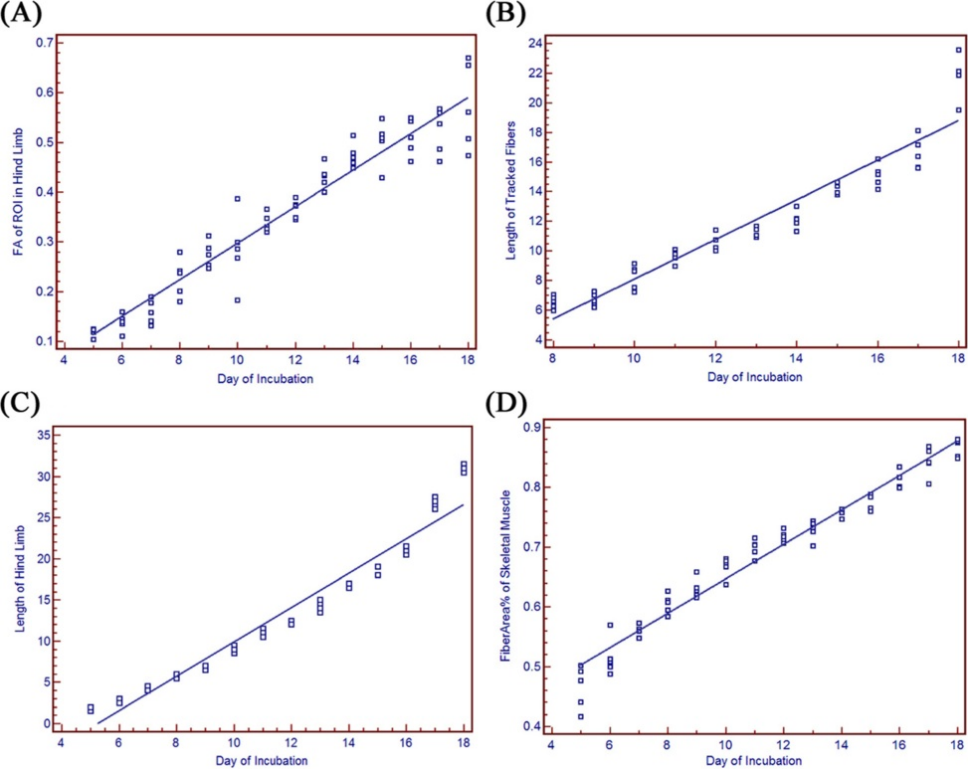
**(A) Relationship between DTI-derived fractional anisotropy (FA) and incubation day. (B)** Relationship between length of tracked fibers in DTI and incubation day. **(C)** Relationship between length of hind limb and incubation day. **(D)** Relationship between histology-derived FiberArea% and incubation day. For all figures, the trend line indicates the result of linear regression analysis.

We were able to discriminate and draw a region of interest (ROI) on the hind limb or hind limb bud on T2 weighted images from day 5. Beginning with day 8, skeletal muscle fibers of the hind limb could be tracked with DTI and the fiber length derived. As shown in Table 1 and Figure 1(A), (B) and (C), fractional anisotropy (FA), tracked fiber length, and hind limb length show a progressive linear increase with each successive day of incubation. The skeletal muscle fibers of the hind limb did not show histologic formation until day 8 or 9, the point at which the myofibers could be visually discerned (tracked) with DTI. As shown in Table 1 and Figure 1(D), the composition of myofibers also showed a progressive linear increase with increasing incubation time as quantified by the percentage of skeletal muscle fiber area in histology specimens (FiberArea%).

**Table 1:** Skeletal muscle FA, length of tracked fibers, length of hind limb and FiberArea% values for chick embryonic hind limb at each day of incubation

**Incubation Day(X)**	**FA ± s.d.**	**Fiber_length± s.d.(mm).**	**FiberArea% ± s.d.**	**Limb_length± s.d.(mm).**
Day 5	0.119 ± 0.009	‡	0.465 ± 0.032	1.700±0.274
Day 6	0.138 ± 0.018	‡	0.516 ± 0.029	2.800±0.274
Day 7	0.159 ± 0.024	‡	0.561 ± 0.008	4.200±0.274
Day 8	0.228 ± 0.039	6.542 ± 0.425	0.605 ± 0.015	5.900±0.224
Day 9	0.275 ± 0.026	6.708 ± 0.429	0.631 ± 0.015	6.900±0.224
Day 10	0.284 ± 0.073	8.258 ± 0.829	0.667 ± 0.016	9.000±0.354
Day 11	0.338 ± 0.019	9.654 ± 0.425	0.698 ± 0.013	10.900±0.418
Day 12	0.366 ± 0.019	10.502 ± 0.585	0.718 ± 0.008	12.100±0.224
Day 13	0.431 ± 0.024	11.236 ± 0.308	0.729 ± 0.014	14.200±0.570
Day 14	0.474 ± 0.025	12.110 ± 0.611	0.757 ± 0.006	16.800±0.274
Day 15	0.501 ± 0.044	14.266 ± 0.381	0.776 ± 0.012	18.200±0.447
Day 16	0.511 ± 0.037	15.094 ± 0.769	0.814 ± 0.013	21.100±0.418
Day 17	0.523 ± 0.047	16.576 ± 1.078	0.844 ± 0.021	26.800±0.570
Day 18	0.573 ± 0.088	21.828 ± 1.454	0.867 ± 0.013	30.900±0.418
**Model Diagnostics**				
n	70	55	70	70
R^2^	0.929	0.908	0.962	0.951
F value	885.825	520.369	1696.561	1313.533
P value	<0.001	<0.001	<0.001	<0.001
Regression Equation	Y_1_ = −0.070 + 0.037 X	Y_2_ = −5.331 + 1.339 X	Y_3_ = 0.357 + 0.029 X	Y_4_= −10.999+ 2.084 X

Model diagnostics show linear regression parameters for incubation day (X) dependence of skeletal muscle FA (Y1), length of tracked fibers(Y2), FiberArea% (Y3) and length of hind limb(Y4) values. ‡: No fibers tracked in DTI from day 5 to 7.

**Figure 2:**
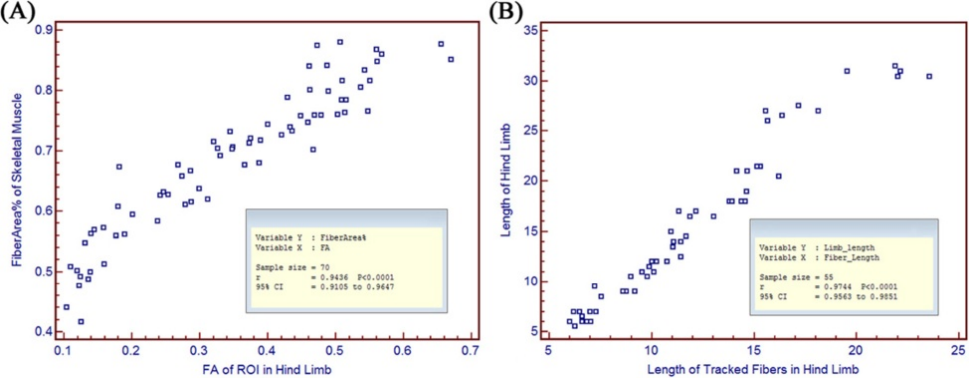
**The result of correlation analysis between parameters in DTI and in histology. (A)** FA and FiberArea%. **(B)** Length of trackd fibers and length of hind limb.

The result of correlation analysis between FA and FiberArea%, and between length of tracked fibers (Fiber_length) and length of hind limb (Limb_length) is shown in Figure 2. FA and FiberArea% have good correlation (correlation coefficient r =0.943, p<0.0001), as do fiber length and limb length (r=0.974, p<0.0001).

**Figure 3:**
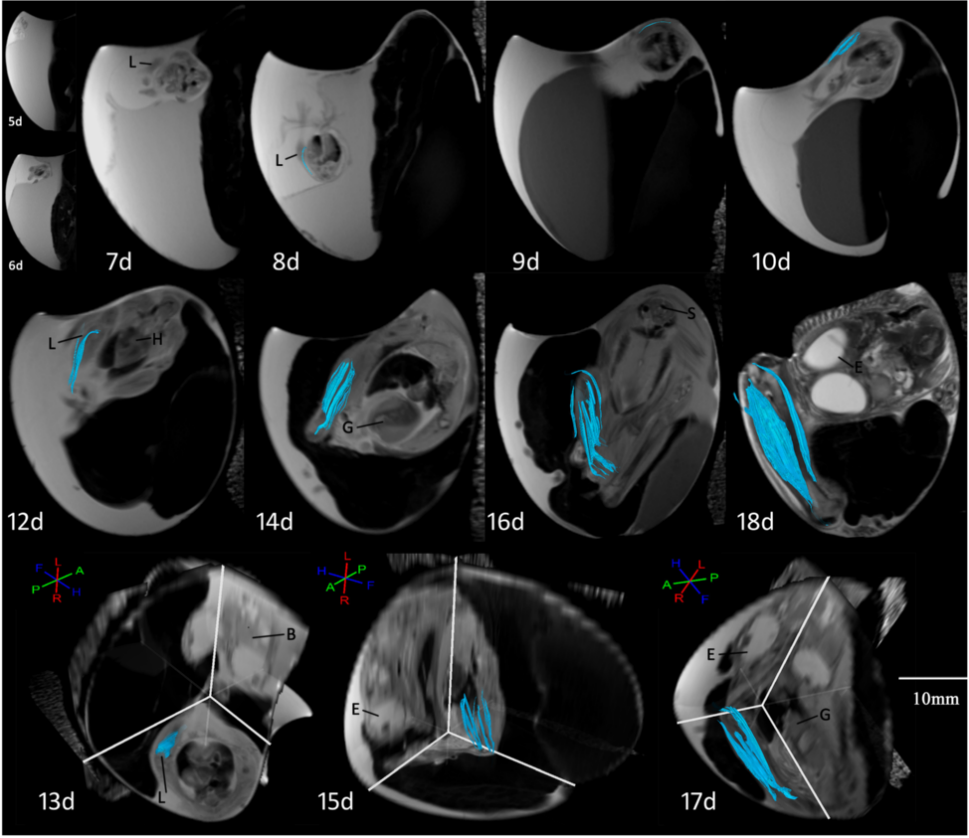
**Skeletal muscle fiber tracking result of chick embryonic hind limb from day 5 of incubation serially.** DTI tracking of muscle fibers (light blue) was successful beginning from day 8. Three dimensional images are included for reference for days 13, 15, and 17. Key: B: brain, E: eye, G: gizzard, H: heart, L: hind limb, S: spine.

**Figure 4:**
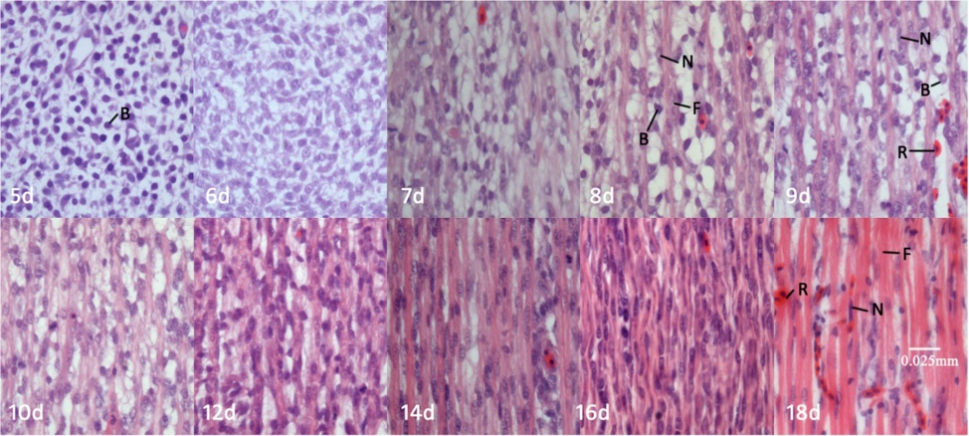
**Histologic specimens of chick embryonic hind limb from day 5 of incubation (400×).** Key: B:myoblast, F:skeletal muscle fiber, N:nucleus, R:red blood cell.

A single typical example of serially DTI-derived skeletal muscle fiber tracking is shown in Figure 3, and shows that skeletal muscle fiber length and number increases with each successive day. Histologic specimens beginning with incubation day 5 are shown in Figure 4.

## Discussion

Our data show that DTI reflects skeletal muscle fiber formation and growth in hind limb during the incubation period. In contrast to invasive methods of analysis, repeated observations on a single embryo throughout incubation can be implemented using DTI and subsequent phenotypic characteristics would be evident at hatching.

Chick embryo skeletal muscle development has been investigated in detail by developmental biologists [13-15]. Multinucleated skeletal myofibers which contain cross-striated myofibrils are formed by the cytoplasmic fusion of mononucleated myoblasts. The myogenic precursor cells delaminate from dermatomyotome of the paraxial mesoderm and migrate to the limb bud, which then proliferate and differentiate into myoblasts. Embryonic myoblasts, which are most abundant on day 5 and disappear on days 7–8, have short fibers and contain few (2 to 3) nuclei; these were unable to be tracked with DTI in this study. Myoblast fiber length and density increase as embryonic myoblasts mature into fetal myoblasts (peaking at days 8–12) which contain hundreds of nuclei, and then later mature into adult myoblasts prior to hatching[14,16,17]. Adult myoblasts located below the basal lamina of adult muscle appear at the late fetal stage and can be activated to divide, differentiate, and form new muscle fibers following injury in postnatal life [16,17]. DTI tracking of fetal and adult stage myoblasts was successful, and progressive increase in myofibril length is evident on fiber tracking images.

The tracked fiber length of the hind limb in DTI was not entirely consistent with the length of hind limb. For this study, we used a simple measurement method, the length of tracked fibers, which is an average value and it is affected by the tractography stopping threshold parameters (minimum FA, maximum angle change, and minimum fiber length). Therefore, while the DTI measured tracking lengths are not necessarily representative of a single myofiber, it can be considered to be a useful relative surrogate given the correlation obtained between Fiber_length and Limb_length.

This study has several limitations. The spatial resolution of 3.0 T MRI limits evaluation of small embryonic structures, particularly during the early incubation period (before 5 days); spatial resolution would almost certainly be improved with a higher field strength magnet. Matching of MRI imaging planes and histologic specimens was performed with careful attention to detail but acquiring exact matches is technically challenging and exact matching was not feasible in all cases. The reverse method, used by some researches, would be to use reconstructions of the isotropic or nearly-isotropic 3D MRI dataset to match the histological sections, possibly obtaining more accurate imaging-histologic correlation. Our dual-cooling method, which reduced the temperature, may influence diffusion measurements (such as apparent diffusion coefficient and mean diffusion) compared to room temperature; we intend to explore this possibility in our next work. Furthermore, there may be developmental differences among embryos of the same incubation day, though this could be accounted for by increasing statistical power.

Given that DTI has the ability to evaluate skeletal muscle differentiation and development, it may be possible to monitor the effect of mutations which affect skeletal muscle migration and development [18,19]. DTI also has the potential to observe the embryonic development of other fiber-like tissues, such as smooth muscle, myocardium and nerves. In particular, DTI has the potential to provide information about white-matter tract integrity within the developing brain and spinal cord. For example, regional dysmyelination effects were able to be measured with DTI within shiverer mouse mutant brain tissue [20], and DTI diffusion parameters showed changes in rodent spinal cords receiving moderate T7 level injury [21]. Similar effects may be possible to measure within the chick embryo.

## Conclusions

In conclusion, this study shows that a widely-available (3.0 T) MRI system can be used as a powerful investigative tool to evaluate embryonic development with diffusion tensor imaging in ovo from 5 days incubation to hatching. We show that acquired skeletal muscle diffusion tensor imaging parameters correlate with histologic specimens from sacrificed embryos. Noninvasive evaluation of developmental processes in embryonic models has traditionally been technically difficult and largely restricted to anatomic evaluation. Functional imaging with DTI MRI and other advanced MRI techniques permit the evaluation of tissues and cellular processes below the conventional spatial resolution of MRI, and is a potentially promising method of improving the understanding of early developmental processes.

## Methods

### Animals and treatments

The experimental protocol and procedures were approved by the Institutional Ethics Committee of the Shanghai Jiaotong University School of Medicine. Ninety (90) fertile Hy-Line White eggs each weighing 50–55 g were obtained from a commercial hatchery and placed in a digital tabletop incubator with temperature (37.8°C) and humidity (60%) controlled automatically. After four days of incubation, eggs were “candled” (using a hand-held light source, light was shone through the egg) to determine if they were fertile and developing normally. Six eggs were removed from the incubator for underdevelopment. From the 84 remaining eggs, 5 eggs were chosen at random each day, from incubation day 5 to 18 (14 days total). Eggs were first removed from the incubator and air-cooled for one hour at 3.5-4°C prior to imaging. The surface temperature of the egg before air-cooling was 24-26°C. During imaging, the egg was wrapped in a single piece of Techni-Ice (Techni Ice, Victoria, Australia). Techni Ice egg-contact surface temperature was 0-2°C, as measured immediately prior to and after imaging. Temperature monitoring during imaging was not performed. The dual-cooling method was used to suppress embryonic motion throughout the imaging session (about 32 minutes). In order to show the in ovo serial skeletal muscle diffusion tractography of the developing chick embryo using DTI, an additional 3 eggs were imaged serially without making histological specimen from day 5 to 18.

### MR Image acquisition

Eggs wrapped in Techni-Ice were imaged in a 3 T Philips Achieva (Philips Medical Systems, Best, Netherlands) using a four-channel dedicated animal coil with a 5 cm inner diameter. Image acquisition consisted of high-resolution T2 weighted SENSE TSE (turbo spin echo) images (accelerated with SENSE, TR/TE=4375/80 ms, FOV 50 × 45 × 42 mm, ETL=13, NEX=10, matrix 250 × 225 (0.2 × 0.2 mm), slice thickness 1.2 mm, no gap, 12 min 23 s duration), and SENSE DTI using SE (spin echo)-DWI (15 directions, TR/TE=5517/65 ms, FOV 50 × 45 × 42 mm, matrix=83 × 75 (0.6 × 0.6 mm), b=500 s/mm2, NEX=2, slice thickness 1.2 mm, no gap, 20 minute duration). A 16th unweighted (b=0) DTI image was also acquired. All imaging planes were sagittal.

### MR fiber tracking

Skeletal muscle fibers in the hind limb were tracked using FiberTrak software (Philips Medical Systems, Best, Netherlands). Three parameters, minimum fractional anisotropy (FA), maximum angle change, and minimum fiber length needed to be set to determine the stopping threshold of tractography. We chose a small minimum FA (0.15) and small maximum angle change (27°) to balance the sensitivity of myofiber tracking during early development stage and the precision during later development; these values were held constant throughout the incubation period. Small values for minimum fiber length (5 mm) were used during early incubation (days 5–11), and minimum fiber length was increased to 10 mm during later incubation (days 12–18).

**Figure 5:**
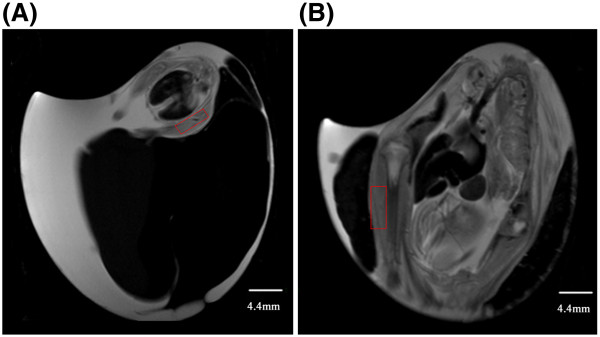
**Example of a rectangular region of interests (ROI) used for DTI fiber tracking. (A)** At incubation day 9, the ROI (red rectangle) covers the entire hind limb bud of the embryo. **(B)** At incubation day 17, the ROI (red rectangle) covers the middle portion of the hind limb, adjacent to the bone.

T2-weighted images were used to guide placement of the rectangular region of interest (ROI) for fractional anisotropy (FA) measurement. The sagittal image containing the largest section of a single hind limb was selected. An ROI was then placed on the hind limb. ROI size changed with embryonic development. As shown in Figure 5, prior to day 10 of incubation, the ROI covered the entire hind limb bud. After day 10 of incubation, the ROI covered the middle portion of the hind limb bud adjacent to, but not including, bone.

### Histology

**Figure 6:**
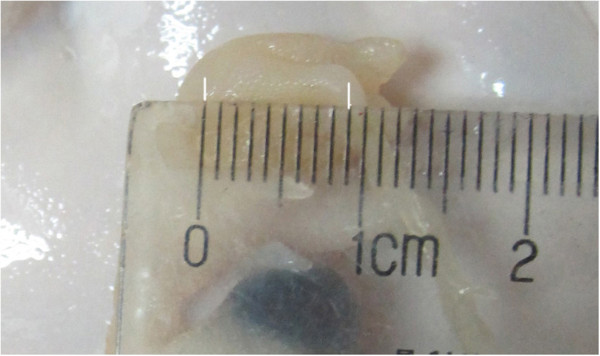
**A simple length measurement of a day 10 chick embryonic hind limb by a ruler.** (about 9 mm).

**Figure 7:**
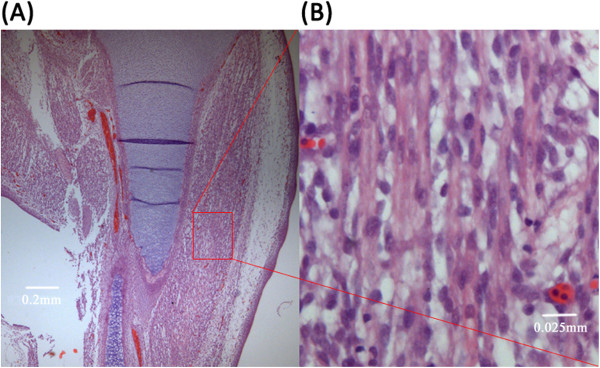
**Example of a region chosen for histologic sampling. (A)** Chick embryonic hind limb at incubation day 9. **(B)** Micrograph (400×) of the region of interest (outlined by the red rectangle) shown in **(A)**.

After MR imaging, the egg was cracked and the corresponding embryonic hind limb bud or hind limb was resected as a specimen and fixed in a 10% formaldehyde solution for one week. The length of hind limb bud or hind limb was simply measured by a ruler before resection. As shown in Figure 6, the length of a 10 day chick embryonic hind limb is about 9 mm. Following dehydration and paraffin embedding, the specimen was serially sectioned at 3 μm in a plane corresponding to the sagittal MR imaging plane. Sections were then stained with hematoxylin and eosin. Each section was observed with the light microscopy (Olympus BX51, Olympus, Tokyo, Japan) and the block region most closely corresponding to the DTI ROI was chosen and micrographed (400× magnification, as shown in Figure 7).

### Data analysis

**Figure 8:**
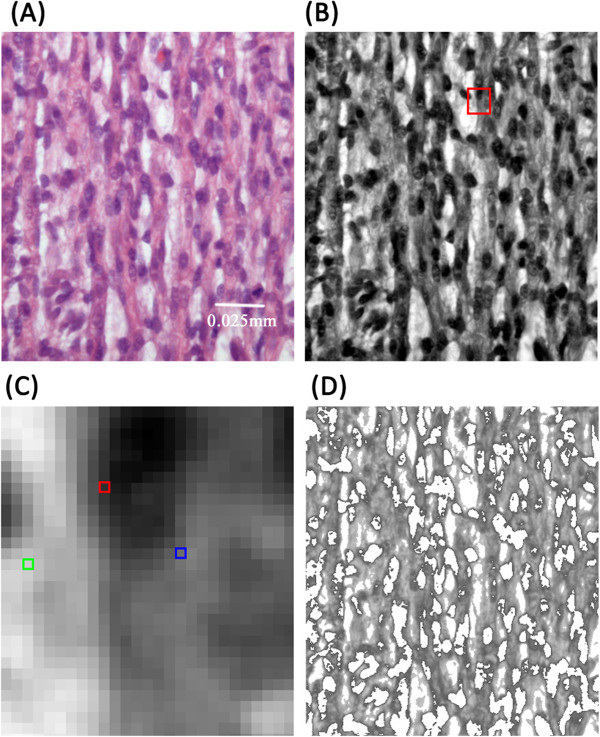
**Illustration of the FiberArea% algorithm. (A)** Chick embryonic hind limb skeletal muscle micrograph (400×) at incubation day 12. **(B)** The corresponding micrograph, converted to grayscale. **(C)** The magnified region of interest (red rectangle) in **(B)**. Example pixel values are: green rectangle, 204, corresponding to background/mesenchymal tissue; red rectangle, 41, corresponding to nucleus; and blue rectangle, 118, corresponding to a myofiber. **(D)** Pixels belonging to the skeletal muscle fibers only (pixels with values between 90 and 200); all the other pixels value is set to white (value 255).

Fractional anisotropy (FA) value and tracked fiber length of tissue within ROI was automatically computed by the FiberTrak software. The percentage of skeletal muscle fiber area (termed FiberArea%) was determined using custom software based on the Insight Segmentation and Registration Toolkit (Kitware Inc., Clifton Park, NY). The algorithm used for calculation of FiberArea% is based on grey-level differences. Color specimen micrographs were converted to an 8-bit grey-scale pictures, with each pixel measuring from 0 (black) to 255 (white). Pixels were classified via thresholding and binning; pixel values below 90 were associated with nucleus or myoblast structures, and values above 200 were associated with background mesenchymal tissue. Pixel values between 90 and 200 were considered to represent skeletal muscle fiber. FiberArea% represents the ratio of binned pixels representing skeletal muscle fiber relative to the total number of pixels within the micrograph. An example is shown in Figure 8.

Data management and statistical analysis was performed using MedCalc (MedCalc Software, Mariakerke, Belgium). A linear model was used to evaluate the relationship between day of incubation and ROI-derived fractional anisotropy (FA) values, length of tracked fibers, FiberArea% and length of hind limb. To evaluate the relationship between imaging and histology during embryonic development, two correlation analyses were performed with p values < 0.05 considered to be significant. One was between FA and FiberArea%, the other was between length of tracked fibers (Fiber_length) and length of hind limb (Limb_length).

## Abbreviations

MRI: Magnetic resonance imaging; DTI: Diffusion tensor imaging; FA: Factional anisotropy; ROI: Region of interest; FiberArea%: The percentage of skeletal muscle Fiber within a given histologic specimen.

## Competing interests

The authors declare that they have no competing interests.

## Authors’ contributions

Research idea and study design, JRX JNH EMH, Chick embryo hatching, ZEZ, MRI acquisition and measurement of diffusion parameters, ZEZ JH YQY ZSZ, Histological specimen making and analysis, LMW, Statistical analysis, ZEZ LMW JRX JNH ZD YZ, Manuscript drafting, ZEZ JH ZD YZ, Manuscript revision for important intellectual content, all authors. Approval of final version of submitted manuscript, all authors. All authors read and approved the final manuscript.
